# Activation of glucagon‐like peptide‐1 receptors and skilled reach foraging

**DOI:** 10.1111/adb.12953

**Published:** 2020-08-08

**Authors:** Jesper Vestlund, Filip Bergquist, Valentina Licheri, Louise Adermark, Elisabet Jerlhag

**Affiliations:** ^1^ Department of Pharmacology, Institute of Neuroscience and Physiology The Sahlgrenska Academy at the University of Gothenburg Gothenburg Sweden; ^2^ Addiction Biology Unit, Department of Psychiatry and Neurochemistry, Institute of Neuroscience and Physiology The Sahlgrenska Academy at the University of Gothenburg Gothenburg Sweden

**Keywords:** electrophysiology, gut‐brain axis, Montoya staircase, motivated behaviours, nucleus accumbens shell

## Abstract

Glucagon‐like peptide‐1 receptor (GLP‐1R) agonists, such as exendin‐4 (Ex4), liraglutide and dulaglutide, regulate glucose homeostasis and are thus used to treat diabetes type II. GLP‐1 also contributes towards a variety of additional physiological functions, including suppression of reward and improvement of learning. Acute activation of GLP‐1R in the nucleus accumbens (NAc) shell, an area essential for motivation, reduces the motivation to consume sucrose or alcohol when assessed in a simple motor task. However, the effects of repeated administration of the different GLP‐1R agonists on behaviours in a more complex motor task are unknown. The aim was therefore to investigate the effects of repeated Ex4, liraglutide or dulaglutide on the motivation and learning of a complex motor tasks such as skilled reach foraging in the Montoya staircase test. To explore the neurophysiological correlates of the different GLP‐1R agonists on motivation, *ex vivo* electrophysiological recordings were conducted. In rats with an acquired skilled reach performance, Ex4 or liraglutide but not dulaglutide reduced the motivation of skilled reach foraging. In trained rats, Ex4 infusion into NAc shell decreased this motivated behaviour, and both Ex4 and liraglutide supressed the evoked field potentials in NAc shell. In rats without prior Montoya experience, dulaglutide but not Ex4 or liraglutide enhanced the learning of skilled reach foraging. Taken together, these findings indicate that the tested GLP‐1R agonists have different behavioural outcomes depending on the context.

## Graphical abstract

The glucagon‐like peptide‐1 receptor (GLP‐1R) agonists, exendin‐4 (Ex4) and liraglutide (Lir) reduces the motivation to skilled reach foraging in rats that have acquired this behaviour, whereas dulaglutide (Dul), enhances the learning of this behaviour in rats without prior experience.

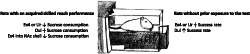

## 1 INTRODUCTION

Glucagon‐like peptide‐1 (GLP‐1) is well known to regulate the blood glucose levels through its ability to increase insulin secretion^1^ and to decrease glucagon excretion.^2^ These physiological findings have led to clinical trials and the subsequent approval of GLP‐1 receptor (GLP‐1R) agonists, such as exendin‐4 (Ex4), liraglutide and dulaglutide, for treatment of diabetes type II.^3^, ^4^, ^5^, ^6^, ^7^, ^8^, ^9^ These GLP‐1R agonists have different pharmacokinetic properties, such as difference in half‐life, which are accounted for when selecting dose and dose interval in patients.^4^, ^5^, ^6^, ^7^, ^9^ Another pharmacokinetic property that differs between GLP‐1R agonists is the distribution volume in patients.^4^, ^5^, ^6^, ^7^, ^9^ In addition, preclinical studies have shown that these agonists have differential ability to penetrate and activate brain regions.^10^, ^11^, ^12^ Although it remains to be studied, these divergent characteristics may contribute to diverse physiological processes modulated by the different GLP‐1R agonists.

Beside glucose homeostasis, GLP‐1 is reported to contribute to a variety of additional physiological processes, including suppression of appetite, where it decreases both homeostatic and hedonic feeding (for review, see Kanoski et al^13^). Moreover, activation of GLP‐1R enhances learning processes in cognitive impaired rodents tentatively by increasing neurogenesis and neuroplasticity.^14^, ^15^, ^16^, ^17^, ^18^ Also, GLP‐1R activation improves reference memory, and augment associative and spatial learning in normal rodents.^16^, ^17^ Reinforcement is another function that GLP‐1 modulates. This is evident because GLP‐1R agonists attenuate the acute rewarding effects of addictive drugs, reduce alcohol consumption and decrease operant self‐administration of alcohol in rodents (for review, see Jerlhag^19^). Further, acute systemic administration of GLP‐1R agonists reduces the operant self‐administration of sucrose and palatable food in rats.^20^, ^21^ These effects are directly connected to an acute activation of GLP‐1R within the nucleus accumbens (NAc) shell,^21^, ^22^ an area crucial for motivated behaviours.^23^ However, the impact of GLP‐1R activation for the motivation and learning of a complex motor behaviour, such as skilled reach foraging, is to date unknown.

The Montoya staircase test measures skilled reach foraging for sucrose pellets,^24^ where the difficulty of ipsilateral retrieval of sucrose pellets from descending steps progressively increases. As the number of pellets consumed and the success rate can be measured in rats with or without prior training, both the motivation in trained rats and the motivation and learning during the acquisition of the task in rats without prior training can be assessed.^24^, ^25^ We therefore used the Montoya staircase test to evaluate the hypothesis that GLP‐1R activation reduces the motivation of skilled reach foraging in trained rats via NAc shell, whereas it increases the learning of such behaviour during behaviour acquisition. Given the above‐mentioned variation in characteristics of Ex4, liraglutide and dulaglutide,^4^, ^5^, ^6^, ^7^, ^9^, ^10^, ^11^, ^12^ we further postulate that these different GLP‐1R agonists may affect the assessed behaviours differently. Therefore, we first evaluate the effects of repeated administration of Ex4, liraglutide or dulaglutide on the motivation of skilled reach foraging in rats with an acquired skilled reach performance. To establish a link between GLP‐1R in NAc shell to the motivation of skilled reach foraging, the influence of local infusion of Ex4 into NAc shell on behaviours was studied. To further explore this NAc‐GLP‐1 link in rats with an acquired skilled reach performance, *ex vivo* electrophysiology was performed during perfusion of GLP‐1R agonists to NAc shell slices from rats with an acquired skilled reach performance. The Montoya staircase test was thereafter used to investigate the effects of repeated systemic administration of Ex4, liraglutide or dulaglutide on the learning of skilled reach foraging in rats without prior Montoya experience. Together, these experiments will define the role of GLP‐1R activation on different aspects of complex motor behaviours.

## 2 MATERIAL AND METHODS

### 2.1 Experimental procedure

A combination of behavioural assessments and electrophysiological recordings was used (Table S1). The motivation of skilled reach performance, as measured by the number of pellets consumed in the Montoya staircase test, was investigated in rats with an acquired skilled reach performance. The Montoya staircase test was also used to assess the learning of skilled reach foraging, that is, the increase in number of pellets consumed and the improvement of success rate, in rats without prior Montoya experience. Field potential and whole cell recordings were conducted to evaluate the effects of GLP‐1R agonist on NAc shell neurotransmission in rats with an acquired skilled reach performance. The rotarod test was used to assess gross motor performance.

### 2.2 Animals

Male Wistar Rcc Han rats (160–190 g; Envigo, Horst, Netherlands) were either housed in groups of four (*n* = 169, in experiments with systemic administration) or individually (*n* = 47, in the experiment with local injections). This strain was used as it exhibits a robust ability to pick sucrose pellets in the Montoya staircase.^25^, ^26^ The rats had free access to water and were kept on a 12/12 h light/dark cycle at 20°C with 50% humidity. Food availability was restricted during night (80% of the daily free‐feeding, which are 8% of their body weight, in chow per day) to enhance the motivation for skilled reach performance.^25^, ^26^ All behavioural experiments were conducted during the light time cycle and rats were only used in one experiment. The Gothenburg Animal Research Ethics Committee approved each experiment (151‐2015).

### 2.3 Drugs

Ex4 (Tocris Bioscience, Abingdon, United Kingdom) was either administered intraperitoneally (IP) or locally into NAc shell 10 min prior to initiation of the experiment.^20^, ^22^, ^27^, ^28^, ^29^, ^30^ The selected IP (1.2 μg/kg, dissolved 0.9% NaCl) or NAc shell (0.05 μg in 0.5 μl per side, dissolved in Ringer solution) dose of Ex4 was used as they reduced reinforcement, without affecting the gross motor behaviour or the kaolin intake in rats.^20^, ^22^, ^27^, ^28^, ^29^, ^30^ Higher doses of Ex4 influence gross behaviour^20^, ^21^, ^30^ and were therefore not used herein. Ex4 was injected IP as IP‐Ex4 reduces alcohol intake in rats^30^ and as IP‐Ex4 has similar latency to effect on feeding as SC‐Ex4.^31^ The selected liraglutide (Novo Nordisk, Kronans Apotek, Gothenburg, Sweden) dose [0.1 mg/kg, subcutaneous (SC)] was chosen as a dose–response study demonstrated that repeated SC injections with this dose as it decreased voluntary alcohol consumption without affecting the rats' gross behaviours.^32^ Although SC‐liraglutide, compared with IP‐liraglutide, increased the latency to effect on feeding,^31^ we still selected the SC route based on the data from the dose–response study^32^ and to mimic the administration route that is used when treating patients.^3^, ^8^

Liraglutide was also administered twice (48 and 24 h) prior to the Montoya staircase test, as this protocol reduces the probability of malaise.^32^ Dulaglutide (0.1 mg/kg, SC; Lilly, Kronans Apotek) was administered once weekly at 48 h prior to the Montoya staircase test. The dose and administration route were selected as a pilot dose–response study shows that repeated injections with this dose reduced alcohol consumption in rats without affecting locomotor activity per se (pilot data). Although dulaglutide is injected twice weekly in other rat studies,^33^, ^34^ dulaglutide was injected once weekly herein based on the pilot study (pilot data) and that others have shown that the plasma levels of dulaglutide are sustained 6 days after acute injection to rats.^33^ In addition, this dose and injection interval mimics the dosing pattern used in patients.^8^ Lower doses of either liraglutide or dulaglutide have less apparent effect on alcohol intake in these studies, whereas higher doses cause tolerance or an effect per se on gross behaviour (pilot data^32^). In the electrophysiology experiments, the selected concentrations of Ex4 (10 nM), liraglutide (1 μM) or dulaglutide (0.1 μM) were used because these roughly correspond to the doses used in the behavioural tests. As low volumes of liraglutide and dulaglutide were dissolved in vehicle (0.9% NaCl), the excipients in these are less likely to be a major contributor to the final dilution.

### 2.4 Guide implantation

The surgery was conducted as earlier described,^25^ where two guides (stainless steel, length 10 mm, with an o.d./i.d. of 0.6/0.45 mm) targeting the NAc shell were implanted 2 days prior to the drug challenge. During surgery, the rats were kept on a heating pad to prevent hypothermia. The rats were anaesthetized with isoflurane (Isofluran Baxter, Kronans Apotek) and a local anaesthetic mixture [Xylocain (10 mg/ml) together with adrenalin (5 μg/ml), Astra Zeneca, Kronans Apotek] was applied on the skull surface. After incision, the skull bone was exposed, and three holes were drilled: two for the guides^35^(Table S2) and one for the anchoring screw. The tips of the guides were inserted 1 mm below the skull bone. The guides were anchored to the screw and the skull bone with dental cement (DENTALON®plus; Agntho's AB, Lidingö, Sweden). Carprofen (5 mg/kg, SC, Rimadyl**®**; Zoetis, Kronans Apotek) was used to relieve pain following surgery.

At the experimental day, a dummy cannula was carefully inserted and retracted into the guide to remove clotted blood and hamper spreading depression within NAc shell. One hour later, a cannula delivered the drug into the NAc shell (Table S2), the drug was delivered over 1 min, and after an additional minute, the cannula was retracted.

### 2.5 Montoya staircase test

The Montoya staircase test was performed as described earlier.^24^, ^25^, ^26^ Three sucrose pellets (45 mg; BioServ, Frenchtown, NJ, USA) were placed on each staircase of the Montoya apparatus (9 × 6 × 30 cm, Campden Instruments Ltd; Loughbrough, United Kingdom) in a sound‐attenuated and ventilated cupboard. At each test day, one rat at the time was placed inside the Montoya apparatus and the rat was allowed to forage for sucrose pellets for 15 min. At each 15‐min session (1–10), the number of sucrose pellets consumed, and the success rate was calculated {success rate = [consumed pellets/(consumed pellets + dropped pellets)]*100}. There was two Montoya test free days between sessions 5 and 6. The drug was either injected to rats with an acquired skilled reach performance or without prior exposure to the Montoya staircase test. In rats with an acquired skilled reach performance alteration of pellets consumed and success rate were measured, as this provides insight into the motivational processes underlying skilled reach foraging (Experiments 1–4). On the other hand, alteration of pellets consumed and success rate in rats without prior exposure to the Montoya staircase test, rather provides insight into motivational and learning processes during acquisition of the task (Experiments 5–7*)* (Table S1).

#### 2.5.1 Treatment effects in rats with an acquired skilled reach performance

Rats were trained in the Montoya staircase and subjected to vehicle treatment for the initial 5 days (Sessions 1–5, baseline) allowing rats to acquire a skilled reach performance.^24^, ^25^ This baseline behaviour was used to divide rats into future treatment groups with similar skilled reach performance. Rats that did not consume any pellets during Sessions 1–5, *i.e.* nonlearners, were excluded (Table S3). A subset of rats was after the initial Montoya training (Sessions 1–5), sacrificed and used in the *ex vivo* electrophysiological tests.

Four separate behavioural experiments were thereafter conducted, where rats received pharmacological treatment or relevant vehicle in combination with the Montoya staircase after Session 5. Thus, rats with an acquired skilled reach performance received vehicle or either Ex4 (IP, Experiment 1), liraglutide (SC, Experiment 2) or dulaglutide (SC, Experiment 3) at Sessions 6–10. In Experiment 3, either vehicle of Ex4 was injected at two additional sessions (Sessions 11 and 12). In the fourth experiment, separate rats were infused with Ex4 or vehicle into the NAc shell (Sessions 6–7).

#### 2.5.2 Treatment effects in rats treated during acquisition of skilled reach performance

Rats without prior exposure to the Montoya staircase test were treated with vehicle or either Ex4 (IP, Experiment 5), liraglutide (SC, Experiment 6) or dulaglutide (SC, Experiment 7). In these experiments, the rats always received treatment in combination with training on the Montoya staircase (Sessions 1–10). Rats that did not consume any pellets during the treatment period (Sessions 1–10) were excluded, as they were nonlearners (Table S3).

### 2.6 Body weight gain

The body weight of each rat was measured each day prior to exposure to the Montoya staircase to calculate the daily amount of food per group‐housed cage needed to get a reliable food restriction and consequently enhance the motivation for skilled reach performance. Thus, the body weight gain (weight on current treatment day/weight at baseline) in percentage (%) was observed, allowing investigation of effects on short‐ (Sessions 6–10) and long‐ (Sessions 1–10) term GLP‐1R agonist treatment on body weight gain.

### 2.7 Rotarod test

All rats were exposed to the rotarod test to achieve groups with similar motor capacities prior to treatment in the Montoya staircase test and to exclude that treatment influences gross motor performance (Table S1). The rotarod test was performed as previous described, and all tests were conducted in the afternoon after the Montoya staircase test.^25^, ^26^ The rotarod device (LE‐8500, Panlab S.L.U.; Barcelona, Spain) was placed in a ventilated and sound attenuating cupboard. The rats were trained to stay on this rotating and accelerating (from 4 to 40 rpm during 5 min) rod for 10 min per session. The average time on the rod of four daily trials was used as an indication of the rat's gross motor performance. The recording of this initial gross motor learning and performance allows a balanced division of rats into future treatment groups (for detailed description, see Table S1). The rats were further tested once a week in the rotarod in the afternoon after the Montoya staircase session in the morning (for detailed description, see Table S1). This defined the effects exhibited by repeated GLP‐1R agonists exposure on gross motor performance.

### 2.8 Electrophysiological recordings

#### 2.8.1 Brain slice preparation

Brain slice preparation for field potential and whole cell recordings was performed as previously described.^36^ Rats were anesthetized, and the brains were removed and submerged in ice‐cold modified aCSF solution containing (in mM); 220 sucrose, 2 KCl, 6 MgCl_2_, 0.2 CaCl_2_, 26 NaHCO_3_, 1.3 NaH_2_PO_4_ and 10 d‐glucose, continuously bubbled with a gas mixture of 95% O_2_/5% CO_2_. Coronal brain slices (250 μm) were transferred to conventional aCSF containing (in mM); 124 NaCl, 4.5 KCl, 2 CaCl_2_, 1 MgCl_2_, 26 NaHCO_3_, 1.2 NaH2PO_4_ and 10 d‐glucose, continuously bubbled with a gas mixture of 95% O2/5% CO_2_. Slices were incubated in aCSF for 30 min at 30°C and then in room temperature for the remainder of the day.

#### 2.8.2 Field potential recordings

*Ex vivo* field potential recordings were performed to investigate acute effects on accumbal neurotransmission elicited by GLP‐1R agonists Ex4 (10 nM), liraglutide (1 μM) or dulaglutide (0.1 μM) in brain slices from animals with an acquired skilled reach performance (Figure S1A). In addition, electrophysiological recordings were also conducted in other brain regions associated with skilled reach performance, including the dorsomedial striatum (DMS; Figure S1A), the dorsolateral striatum (DLS, Figure S1A) and the medial prefrontal cortex (mPFC, Figure S1B).^37^, ^38^, ^39^, ^40^, ^41^ In these experiments, Ex4 (10 nM) was perfused onto brain slices from rats with an acquired skilled reach performance. For field potential recordings, one hemisphere of a slice was transferred to a recording chamber and perfused with prewarmed aCSF kept at 30°C as previously described elsewhere.^36^ Population spikes (PS) were activated by paired pulse stimulation (50 ms interpulse interval) at a frequency of 0.05 Hz. Stimulus intensity (0.01–0.04 mA) was adjusted so that the PS amplitude was approximately half the size of the maximal response. Signals were amplified by a custom‐made amplifier, filtered at 3 kHz, and digitized at 8 kHz. Following a stable baseline, the drugs were administered via the perfusion system. This allowed us to investigate the acute effect of the drug on PS amplitude in the NAc shell in brain slices from rats with an acquired skilled reach performance.

#### 2.8.3 Whole cell recordings

Whole cell recordings in voltage‐clamp mode were performed to further assess the underlying effects of GLP‐1R activation after perfusion of Ex4 to NAc shell brain slices *ex vivo* from rats with, or without, an acquired skilled reach performance. This method is described in detail elsewhere.^36^ Spontaneous inhibitory currents (sIPSCs) were isolated by blocking NMDA and AMPA receptors using 50‐μM AP5 and 10‐μM CNQX. NAc shell were identified using a 10×/0.30 objective attached to a Nikon Eclipse FN‐1 microscope, whereas a 40×/0.80 water‐immersion objective was used to localize medium spiny neurons (MSNs). Recording pipettes with a resistance ranging from 2.5 to 4.5 MΩ were prepared from borosilicate glass (Sutter Instruments; Novato; CA, USA) and filled with an internal solution containing (in mM): 150 CsCl, 10 Hepes, 2 MgCl_2_, 0.3 Na_2_GTP, 3 MgATP, and 0.2 BAPTA, pH adjusted to 7.2 with CsOH, with osmolarity set to 298 mOsm with sucrose. MSNs were voltage clamped at −65 mV, and a stable baseline response was observed over 5–10 min. Thereafter, Ex4 in prewarmed aCSF (33–34°C, 2 ml/min) were perfused onto the NAc shell brain slices for 10 min and spontaneous activity was recorded. The data used in the analysis were from the last 3 min of bath perfusion. Data were amplified using an Axopatch 700B amplifier (Axon Instruments; Foster City, CA, USA), filtered at 2 kHz, digitized at 5 kHz and acquired using Clampex 10.2 (Molecular Devices, Foster City, CA, USA). Only recordings with a stable series resistance that varied less than 20% and did not exceed 25 MΩ were included in the analysis.

### 2.9 Statistical analysis

The data from the Montoya staircase test, rotarod test and body weight changes were analysed using a repeated measure two‐way analysis of variance (ANOVA) or two‐tailed unpaired *t* test when applicable. Data from electrophysiological recordings were analysed using Clampfit (Molecular Devices, Foster City, CA), Minianalysis 6.0 software (Synaptosoft; Decatur, GA, USA) and Graph Pad Prism. Treatment effects were analysed using repeated measure two‐way ANOVA, or two‐tailed paired *t* test when applicable. All data are presented as mean values ± SEM, and the level of significance was set to *P* < 0.05.

## 3 RESULTS

### 3.1 Effects of Ex4, liraglutide and dulaglutide in rats with an acquired skilled reach performance

During training at Sessions 1–5, all rats in Experiments 1–4 increased the number of pellets consumed and the success rate, indicating that all rats established a skilled reach performance (Table S4). In addition, there were no differences in time at the rotarod between groups prior to treatment (Table S4).

#### 3.1.1 Effects of Ex4 on behaviour in the Montoya staircase test and electrophysiological recordings

**FIGURE 1:**
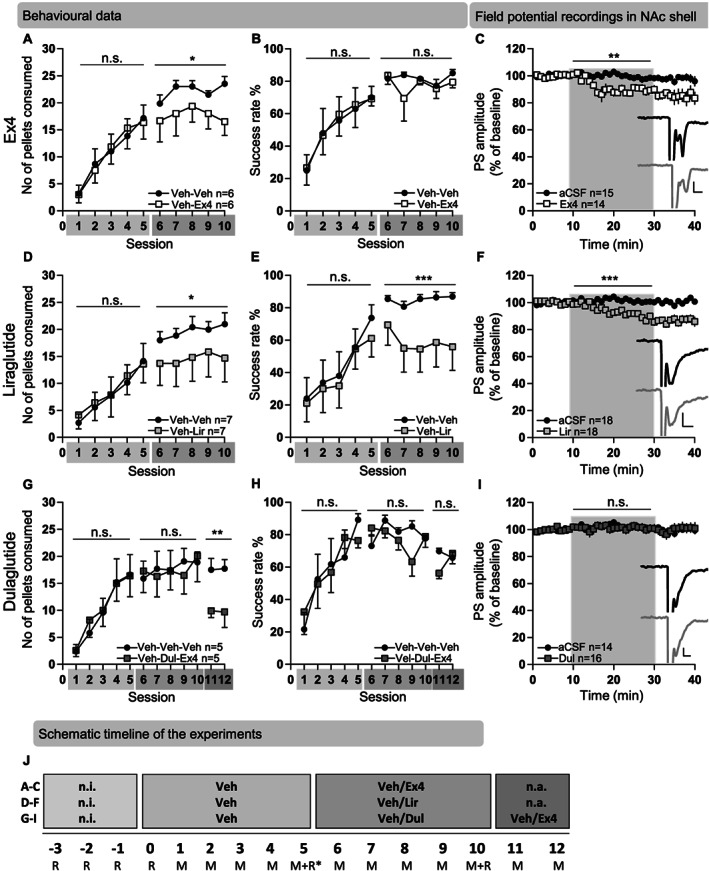
Effects of exendin‐4, liraglutide and dulaglutide on the motivation of skilled reach foraging and *ex vivo* accumbal output in rats with an acquired skilled reach performance. A, Rats established a similar acquired skilled reach performance during the first vehicle (Veh)‐treatment period (Sessions 1–5) as there were no differences in the number of pellets consumed between the future treatment groups (Sessions 1–5). Repeated exendin‐4 (Ex4) treatment decreased the number of pellets consumed in rats with an acquired skilled reach performance compared with Veh (Sessions 6–10). B, There were no differences in success rate during baseline (Sessions 1–5), nor following active treatment in the Ex4 or Veh groups (Sessions 6–10). C, Compared with aCSF, Ex4 infusion suppressed the evoked PS amplitude of NAc shell slices from rats with an acquired skilled reach performance. Light grey square represents when Ex4 or aCSF was perfused into the slices. Example traces showing evoked PS in the NAc shell at baseline (black) and following administration of Ex4 (10 nM; grey). The calibration is 0.2 mV, 2 ms. D, There were no differences in the number of pellets consumed between the future treatment groups during the Veh‐treatment period (Sessions 1–5), showing that rats established a similar acquired skilled reach performance. Repeated liraglutide (Lir) treatment decreased the number of pellets consumed in rats with an acquired skilled reach performance compared with Veh (Sessions 6–10). E, There were no differences in the success rate at baseline between future treatment groups (Sessions 1–5). Repeated injection of Lir suppressed the success rate in rats with acquired skilled reach performance compared with Veh (Sessions 6–10). F, Lir infusion suppressed the evoked PS amplitude of NAc shell slices from rats with an acquired skilled reach performance. Light grey square represents when Lir or aCSF was perfused into the slices. Example traces showing evoked PS in the NAc shell at baseline (black) and following administration of Lir (1 μM; grey). The calibration is 0.2 mV, 2 ms. G, There were no differences in the number of pellets consumed between the future treatment groups (Sessions 1–5) showing that rats established a similar acquired skilled reach performance. Compared with Veh, repeated dulaglutide (Dul) treatment (Sessions 6–10) did not alter the number of pellets consumed in rats with an acquired skilled reach performance. To confirm that Ex4 reduce the consumption of sucrose pellets in rats with an acquired skilled reach performance, Ex4 were injected acutely (Sessions 11–12) to the Dul treated rats. Ex4 reduced the number of pellets consumed compared with vehicle treated rats. H, There were no differences in success rate when it comes to baseline (Sessions 1–5), repeated Dul/Veh treatment (Sessions 6–10) or Ex4/Veh injections (Sessions 11–12). I, Electrophysiological recordings of NAc shell slices from rats with an acquired skilled reach performance revealed that Dul infusion did not alter the evoked PS amplitude compared with aCSF. Light grey square represents when Dul or aCSF was perfused into the slices. Example traces showing evoked PS in the NAc shell at baseline (black) and following administration of Dul (0.1 μM; grey). The calibration is 0.2 mV, 2 ms. J, Schematic timeline of the experiments conducted. Behaviour from these rats, exposed to the rotarod test (R) and Montoya staircase test (M) and treated with either Ex4, Lir, Dul or Veh, are presented in Figure 1A–I. The timeline also shows when a subgroup of rats were euthanized and used for electrophysiological recordings (*). Data are presented as mean ± SEM; ^***^ *P* < 0.001, ^**^ *P* < 0.01, ^*^ *P* < 0.05. n.a., not applicable; , n.i., no injection; n.s., not significant

In comparison to vehicle, repeated Ex4 during Sessions 6–10 reduced the number of sucrose pellets consumed (treatment *F*(1,25) = 6.54, *P* = 0.017, time *F*(4,25) = 0.46, *P* = 0.764, interaction *F*(4,25) = 0.16, *P* = 0.955; Figure 1A), where Ex4 reduced the number of pellets consumed by 16% compared with vehicle at Session 6. Ex4 did not alter the success rate (treatment *F*(1,25) = 1.36, *P* = 0.255, time *F*(4,25) = 0.61, *P* = 0.660 interaction *F*(4,25) = 0.59, *P* = 0.670; Figure 1B). Ex4 did not alter time at the rotarod compared with vehicle treatment (*t*(10) = 0.57, *P* = 0.580), indicating that Ex4 does not alter gross motor performance (Figure S3A). Ex4, administered to rats with an acquired skilled reach performance (Sessions 6–10), did not alter body weight gain (treatment *F*(1,25) = 0.0001, *P* = 0.992, time *F*(4,25) = 41.15, *P* < 0.001, interaction *F*(4,25) = 0.196, *P* = 0.938; Figure S4A), when compared with vehicle.

Perfusion of Ex4 to NAc shell brain slices (*n* = 14) reduced evoked PS amplitude (*F*(1,27) = 12.11, *P* = 0.002; Figure 1C) compared with aCSF treated slices (*n* = 15), suggesting that Ex4 suppresses neurotransmission in NAc shell. Whole cell recordings showed that inhibitory neurotransmission in MSNs of NAc shell was not effected by Ex4 in neither training naive animals (sIPSC amplitude (*t*(4) = 0.64, *P* = 0.557); Figure S2A; sIPSC frequency (*t*(4) = 0.21, *P* = 0.843); Figure S2B), nor in animals with an acquired motor skill (sIPSC amplitude (*t*(6) = 1.97, *P* = 0.096); Figure S2C; sIPSC frequency (*t*(6) = 0.89, *P* = 0.409); Figure S2D).

Electrophysiological recordings were also made on DMS (Figure S1A), DLS (Figure S1A) and mPFC (Figure S1B) slices. Compared with aCSF‐treated baseline, perfusion of Ex4 decreased evoked PS amplitude in DMS (*F*(1,23) = 32.90, *P* < 0.001; Figure S1C), DLS (*F*(1,27) = 48.48, *P* < 0.001; Figure S1D) and mPFC (*F*(1,24) = 21.74, *P* < 0.001; Figure S1E) in slices from rats with acquired skilled reach performance.

#### 3.1.2 Effects of liraglutide on behaviour in the Montoya staircase test and electrophysiological recordings

Compared with vehicle, injections of liraglutide during Sessions 6–10 reduced the number of sucrose pellets consumed (treatment *F*(1,30) = 4.25, *P* = 0.048, time *F*(4,30) = 0.27, *P* = 0.895, interaction *F*(4,30) = 0.03, *P* = 0.999; Figure 1D) and liraglutide suppressed the success rate, reflecting a decreased execution of skilled reach performance (treatment *F*(1,30) = 15.25, *P* < 0.001, time *F*(4,30) = 0.25, *P* = 0.906, interaction *F*(4,30) = 0.17, *P* = 0.954; Figure 1E). Liraglutide did not alter time at the rod compared with vehicle (*t*(12) = 0.96, *P* = 0.357), indicating that liraglutide does not alter gross motor performance (Figure S3B). Liraglutide to rats with acquired skilled reach performance (Sessions 6–10) decreased the body weight gain (treatment *F*(1,30) = 19.05, *P* < 0.001, time *F*(4,30) = 37.18, *P* < 0.001, interaction *F*(4,30) = 0.12, *P* = 0.973; Figure S4B), when compared with vehicle.

Electrophysiological recordings revealed that perfusion of liraglutide to NAc shell brain slices (*n* = 18) reduced evoked PS amplitude compared with aCSF treated slices (*n* = 18) (*F*(1,34) = 24.83, *P* < 0.001; Figure 1F), suggesting that liraglutide suppresses neurotransmission in NAc shell.

#### 3.1.3 Effects of dulaglutide on behaviour in the Montoya staircase test and electrophysiological recordings

Dulaglutide administration during Sessions 6–10 did neither affect the number of sucrose pellets consumed (treatment *F*(1,20) = 0.02, *P* = 0.893, time *F*(4,20) = 0.24, *P* = 0.911, interaction *F*(4,20) = 0.10, *P* = 0.980; Figure 1G) nor alter the success rate (treatment *F*(1,20) = 1.68, *P* = 0.210, time *F*(4,20) = 1.14, *P* = 0.365 interaction *F*(4,20) = 2.64, *P* = 0.064; Figure 1H). However, when rats that were previously treated with dulaglutide instead received two Ex4 injections (Sessions 11–12), the number of sucrose pellets consumed was significantly reduced (treatment *F*(1,8) = 17.07, *P* = 0.003, time *F*(1,8) = 0, *P* = 0.999, interaction *F*(1,8) = 0.01, *P* = 0.918; Figure 1G). Here, Ex4 reduced the number of pellets consumed by 44% compared with vehicle at Session 11. These rats that have acquired the skilled reach behaviour during 10 days before Ex4 administration (number of pellets decreased compared with their own baseline day 10: 10.2 ± 4.2) decreased the consumption of sucrose more profoundly compared with rats that have acquired this skilled reach behaviour during 5 days before Ex4 administration (number of pellets decreased compared with their own baseline day 5: −0.3 ± 2.4) [unpaired *t* test: (*t*(9) = 2.27, *P* = 0.049)].

Like in the previous experiment, Ex4 did not alter the success rate (Sessions 11–12; treatment *F*(1,8) = 1.11, *P* = 0.322, time *F*(1,8) = 2.38, *P* = 0.161, interaction *F*(1,8) = 2.41, *P* = 0.159; Figure 1H). Dulaglutide did not alter time at the rod compared with vehicle (*t*(8) = 0.48, *P* = 0.645) indicating that dulaglutide does not alter gross motor performance (Figure S3C). Dulaglutide administration to rats with acquired skilled reach performance (Sessions 6–10) did not alter the body weight gain (treatment *F*(1,20) = 0.58, *P* = 0.454, time *F*(4,20) = 35.12, *P* < 0.001, interaction *F*(4,20) = 0.12, *P* = 0.974; Figure S4C), when compared with vehicle.

Electrophysiological studies showed that perfusion of dulaglutide (0.1 μM) to NAc shell brain slices (*n* = 16) did not alter evoked PS amplitude compared with aCSF treated slices (*n* = 14) (*F*(1,26) = 0.01, *P* = 0.904; Figure 1I), suggesting that acute administration of dulaglutide does not alter neurotransmission in NAc shell at the concentration used.

#### 3.1.4 Effects of Ex4 into NAc shell on behaviour in the Montoya staircase test

**FIGURE 2:**
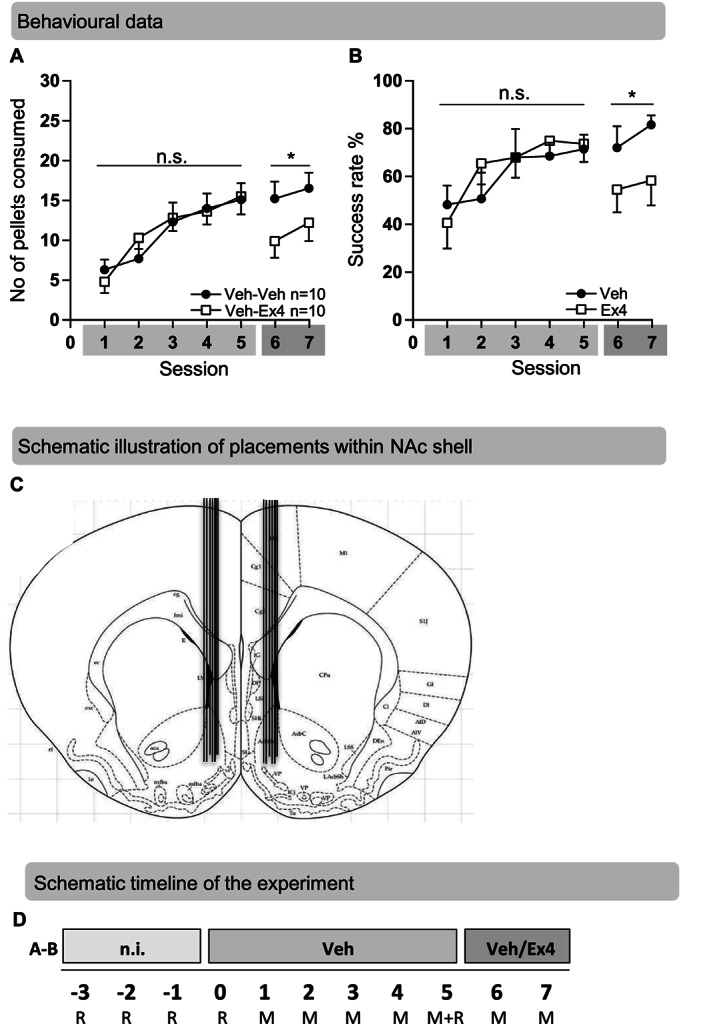
Effects of exendin‐4 into nucleus accumbens shell on the motivation of skilled reach foraging in rats with an acquired skilled reach performance. A, There were no differences in the number of pellets consumed between the future treatment groups, revealing that rats established a similar acquired skilled reach performance during the first vehicle (Veh)‐treatment period (Sessions 1–5). Compared with Veh, exendin‐4 (Ex4) infused into nucleus accumbens (NAc) shell (Sessions 6–7) decreased the number of pellets consumed in rats with an acquired skilled reach performance. B, There were no differences in success rate at baseline (Sessions 1–5) between future treatment groups. In comparison with Veh, Ex4 infused (Sessions 6–7) into NAc shell suppressed the success rate in rats with an acquired skilled reach performance. C, A coronal rat brain section showing seven representative bilateral cannula placements (illustrated by vertical lines) aiming at the NAc shell. D, Schematic timeline of the experiment conducted. Behaviour from these rats, exposed to the rotarod test (R) and Montoya staircase test (M) and infused with either Ex4, or Veh in NAc shell, are shown in Figure 2A–B. Data are presented as mean ± SEM; ^*^ *P* < 0.05. n.i., no injection; n.s., not significant

Infusion of Ex4 into NAc shell for two subsequent days reduced the number of sucrose pellets consumed (treatment *F*(1,18) = 4.66, *P* = 0.047, time *F*(1,18) = 0.78, *P* = 0.390, interaction *F*(1,18) = 0.05, *P* = 0.825; Figure 2A) and decreased the success rate, reflecting suppressed execution of skilled reach performance (treatment *F*(1,18) = 4.63, *P* = 0.045, time *F*(1,18) = 0.78, *P* = 0.389, interaction *F*(1,18) = 0.09, *P* = 0.771; Figure 2B). The infusion sites within NAc shell were verified post mortem (Figure 2C), and no rats were excluded due to misplacements. Ex4 into NAc shell to rats with acquired skilled reach performance (Sessions 6–7) did not alter body weight gain (treatment *F*(1,18) = 0.009, *P* = 0.927, time *F*(1,18) = 3.75, *P* = 0.069, interaction *F*(1,18) = 0.06, *P* = 0.807; Figure S4D).

### 3.2 Effects of Ex4, liraglutide and dulaglutide in rats without Montoya experience

There were no differences in time at the rotarod between groups prior to treatment (Table S4).

#### 3.2.1 Effects of Ex4 on behaviour in the Montoya staircase test

**FIGURE 3:**
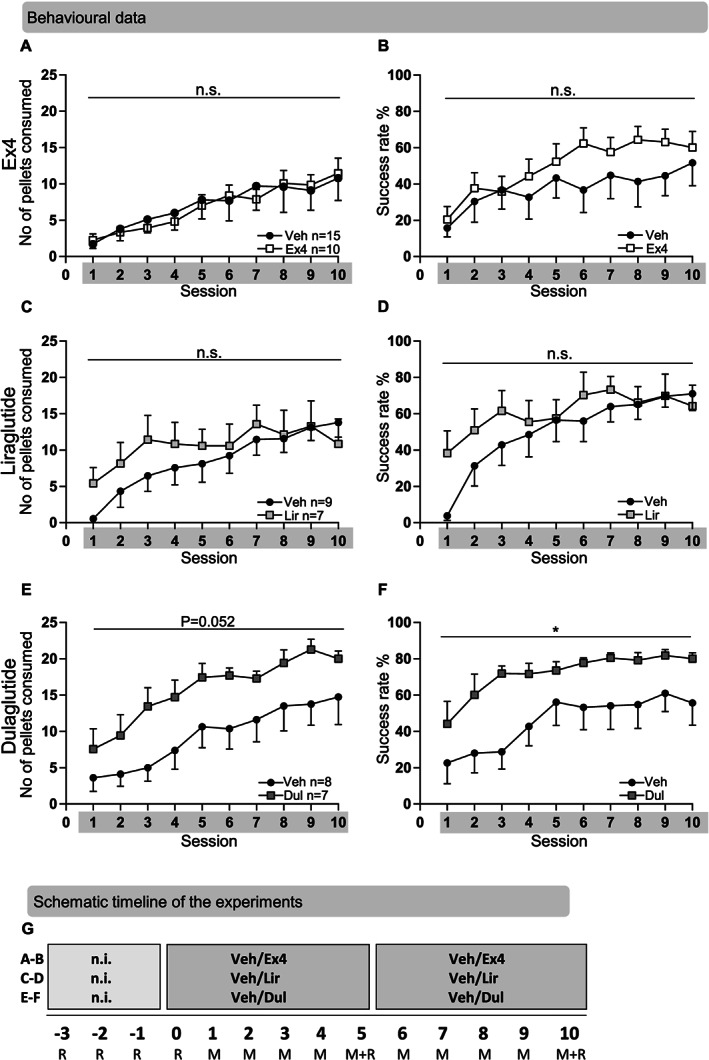
Effects of exendin‐4, liraglutide and dulaglutide on the learning of skilled reach foraging in rats without prior Montoya experience. A, Repeated exendin‐4 (Ex4) treatment did not alter the number of pellets consumed compared with vehicle (Veh) in the Montoya staircase. B, Compared with Veh, Ex4 did not influence the success rate. C, Repeated liraglutide (Lir) treatment did not alter the number of pellets consumed compared with Veh. D, In comparison with Veh, Lir did not influence the success rate. E, Dulaglutide (Dul) treatment while training on the Montoya staircase test had a tendency to increase the number of pellets consumed compared with Veh. F, Dul enhanced the success rate compared with Veh. G, Schematic timeline of the experiments conducted. Behaviour from these rats, exposed to the rotarod test (R) and Montoya staircase test (M) and treated with either Ex4, Lir, Dul or Veh, are presented in Figure 3A–F. Data are presented as mean ± SEM; ^*^ *P* < 0.05. n.i., no injection; n.s., not significant

In rats without prior Montoya staircase experience, repeated Ex4 treatment during Sessions 1–10 did not alter the number of pellets consumed (treatment *F*(1,23) = 0.01, *P* = 0.9225, time *F*(9,207) = 16.55, *P* < 0.001, interaction *F*(9,207) = 0.43, *P* = 0.915; Figure 3A) and did not alter the success rate (treatment *F*(1,23) = 1.08, *P* = 0.309, time *F*(9,207) = 8.23, *P* < 0.001, interaction *F*(9,207) = 0.99, *P* = 0.449; Figure 3B) when compared with vehicle. Compared with vehicle, Ex4 did not influence time at the rotarod (*F*(1,23) = 1.41, *P* = 0.247, time *F*(2,46) = 2.95, *P* = 0.062, interaction *F*(2,46) = 0.70, *P* = 0.503), indicating that Ex4 does not alter gross motor performance (Figure S5A). Repeated Ex4 throughout the entire training period (Sessions 1–10) had no significant effect on the body weight gain (treatment *F*(1,23) = 3.80, *P* = 0.063, time *F*(9,207) = 722.5, *P* < 0.001, interaction *F*(9,207) = 0.95, *P* = 0.483; Figure S6A), when compared with vehicle.

#### 3.2.2 Effects of liraglutide on behaviour in the Montoya staircase test

Compared with vehicle, liraglutide injections during Sessions 1–10 did not alter the number of pellets consumed (treatment *F*(1,14) = 0.52, *P* = 0.484, time *F*(9,126) = 8.60, *P* < 0.001, interaction *F*(9,126) = 1.26, *P* = 0.268; Figure 3C) and did not alter the success rate (treatment *F*(1,14) = 0.78, *P* = 0.392, time *F*(9,126) = 9.21, *P* < 0.001, interaction *F*(9,126) = 1.46, *P* = 0.170; Figure 3D). Compared with vehicle, liraglutide did not influence time at the rotarod (*F*(1,14) = 0.06, *P* = 0.803, time *F*(1,14) = 17.33, *P* = 0.001, interaction *F*(1,14) = 0.001, *P* = 0.972), indicating that liraglutide does not alter gross motor performance (Figure S5B). When it comes to body weight gain, there was no overall effect of treatment (*F*(1,14) = 1.00, *P* = 0.335), but of time (*F*(9,126) = 741 *P* < 0.001) and interaction (*F*(9,126) = 1.99, *P* = 0.045; Figure S6B).

#### 3.2.3 Effects of dulaglutide on behaviour in the Montoya staircase test

Repeated dulaglutide treatment throughout the entire Montoya staircase tests (Sessions 1–10) had a tendency to enhance the number of pellets consumed (*F*(1,13) = 4.57, *P* = 0.052, time *F*(9,117) = 18.61, *P* < 0.001, interaction *F*(9,117) = 0.47, *P* = 0.895; Figure 3E) and increased the success rate (treatment *F*(1,13) = 5.40, *P* = 0.037, time *F*(9,117) = 8.64, *P* < 0.001, interaction *F*(9,117) = 0.72, *P* = 0.690; Figure 3F) in rats without prior Montoya staircase experience. Compared with vehicle, dulaglutide did not influence time at the rotarod (treatment *F*(1,13) = 0.04, *P* = 0.842, time *F*(1,13) = 2.52, *P* = 0.136, interaction *F*(1,13) = 0.03, *P* = 0.856), indicating that dulaglutide does not alter gross motor performance (Figure S5C). Repeated dulaglutide throughout the entire training period (Sessions 1–10) did not affect the body weight gain (treatment *F*(1,13) = 1.61, *P* = 0.227, time *F*(9,117) = 533.2, *P* < 0.001, interaction *F*(9,117) = 0.39, *P* = 0.939; Figure S6C), when compared with vehicle.

## 4 DISCUSSION

The data presented herein suggest that the tested GLP‐1R agonists have different effects on skilled reach behaviours. Indeed, we suggest that Ex4 and liraglutide decrease motivation of skilled reach foraging in rats with acquired skilled reach performance, whereas dulaglutide increases learning of this complex behaviour during acquisition of the task.

The first part of the present study conducted in rats with an acquired skilled reach performance shows that repeated treatment with Ex4 or liraglutide reduces the consumption of sucrose pellets. In addition, liraglutide decreases the success rate without altering gross behaviour on the rod, whereas Ex4 does not alter any of these behaviours. In line, as Ex4 decreases consumption of sucrose pellets and does not alter success rate, it means that the number of pellets dropped also decreases indicating that the motor coordination of the rats is unchanged. We therefore postulate that repeated activation of the GLP‐1R decreases the motivation of skilled reach foraging. These data thereby expand on previous studies demonstrating that acute administration of a GLP‐1R agonist reduces the motivation to consume sucrose, palatable food or alcohol in a simpler motor task, namely theoperant progressive ratio self‐administration task.^20^, ^21^, ^30^, ^32^ Moreover, Ex4 appears to show a more robust inhibitory effect on motivation in rats with more established skilled reach performance. Indeed, Ex4 reduces the number of pellets consumed by 44% in rats that had acquired a skilled reach performance during 10 days before Ex4 treatment and by 16% in rats that had acquired a skilled reach performance during 5 days before Ex4 treatment. These data are in line with the behavioural response of another appetite regulatory peptide, ghrelin, where a ghrelin receptor antagonist suppresses the motivation of skilled reach foraging more profound in rats that have acquired a skilled reach behaviour during more training days.^25^ This collectively suggests that the ability of appetite‐regulatory peptides to reduce the motivation of skilled reach foraging depends on prior acquisition of the task. This is further evident in other reward‐related behaviours, where these gut‐brain peptides reduce alcohol intake in high, but not low alcohol consuming rats.^32^, ^42^, ^43^

The electrophysiological and behavioural findings from the rats with an acquired skilled reach performance together support the notion that GLP‐1R within the NAc shell modulates the motivation of skilled reach foraging. First, the field potential recordings in NAc shell slices reveal that Ex4 or liraglutide suppresses neurotransmission in rats that have acquired the task. Second, infusion of Ex4 into NAc shell decreases the consumption of sucrose pellets. This GLP‐1‐NAc‐motivation link is further supported by the behavioural data from simpler motor tasks demonstrating that acute activation of GLP‐1R in NAc shell not only reduces cocaine‐seeking behaviour,^28^ oxycodone‐seeking behaviour^44^ and alcohol intake^27^, ^29^ but also decreases high‐fat diet intake^22^ and operant progressive ratio self‐administration of sucrose.^21^ The findings that systemic administration of Ex4 reaches the NAc shell^28^, ^44^ further indicate that the NAc shell mechanisms may explain the behavioural outcomes following repeated Ex4‐IP. Behavioural and electrophysiological data from a recent study reveal that NAc shell dependent mechanisms also mediate the ability of a ghrelin receptor antagonist to decrease the motivation of skilled reach foraging.^25^

The whole cell recordings of NAc shell slices from rats with an acquired skilled reach performance show no effect by Ex4 on the frequency or amplitude of inhibitory inputs. Although it remains to be studied in detail, we speculate that Ex4 attenuates motivated behaviours by activating presynaptic GLP‐1R on glutamatergic terminals,^45^ which are coupled to Gα_i/O_ protein associated with decreased excitatory responses.^46^, ^47^ Albeit we here suggest that GLP‐1R activation within NAc shell supresses the motivation for skilled reach foraging in rats with an acquired skilled reach performance, the possibility should be considered that GLP‐1R within other brain regions targeted by systemic administration of GLP‐1R agonists might be involved. For instance, other reward‐related behaviours are supressed by activation of GLP‐1R within ventral tegmental area,^22^, ^27^ laterodorsal tegmental area^27^ and the ventral hippocampus.^48^ Besides NAc shell, the neuronal circuits involved in skilled reach foraging are complex, where projections from the mPFC to the DMS are crucial for the initial learning, and the DLS is important during consolidation of a motor skill.^37^, ^38^, ^39^, ^40^, ^41^ These areas could tentatively be involved in the Ex4‐induced suppression of sucrose pellets consumption by influencing on learning and consolidation of this skilled reach behaviour. In support for this contention are our electrophysiological recordings in brain slices from rats with acquired skilled reach performance showing that perfusion of Ex4 onto DMS, DLS or mPFC decreases neurotransmission in these areas. Upcoming studies should evaluate the individual contribution of GLP‐1R activation in these additional areas for skilled reach performance. In particular, the contribution of GLP‐1R on glutamatergic neurons projecting from the ventral hippocampus to mPFC on skilled reach foraging should be explored as they regulate impulsive operant responding for palatable food.^48^

In rats without prior Montoya experience, repeated treatment with dulaglutide during Sessions 1–10 increases success rate and tends to increase the number of pellets consumed. This enhanced performance, driven by an improvement in success rate, indicates that dulaglutide during acquisition of this complex motor behaviour enhances the learning of skilled reach foraging. A role of GLP‐1 signalling for learning is further supported by the data revealing that activation of GLP‐1R improves reference memory and enhances associative and spatial learning in normal rodents.^16^, ^17^ In addition, the findings that GLP‐1R signalling increases neurogenesis and neuroplasticity in cognitive impaired rodents, which are associated with improved learning and memory function in these rodents,^14^, ^15^, ^16^, ^17^, ^18^ further strengthen the association between GLP‐1R activation and enhanced learning and memory processes. In agreement are the findings that ghrelin signalling enhances performance via increased motivation and learning in rats without prior Montoya experience.^25^ The downstream mechanisms and brain areas responsible for the ability of systemic administration of dulaglutide to enhance learning and consolidation of a motor skill should be evaluated in future studies.

The present behavioural and electrophysiological data reveal that both rats with or without prior Montoya experience respond differently to the tested GLP‐1R agonists. In rats with an acquired skilled reach performance dulaglutide, opposed to Ex4 and liraglutide, does neither affect the motivation to consume sucrose nor alter the neurotransmission in NAc shell slices. This behavioural difference is also evident in rats without prior Montoya experience, where liraglutide or Ex4 in contrast to dulaglutide does not alter learning of this complex motor task. Tentative mechanisms for these outcomes may include diverse capacity to reach deeper brain areas,^10^, ^11^, ^12^ which should be studied in more detail for Ex4 and liraglutide and remains to be fully described for dulaglutide. It may also be associated with a different ability of these agents to influence central neurotransmission,^49^, ^50^ which is supported by the present electrophysiological recordings where liraglutide and Ex4, but not dulaglutide, suppresses neurotransmission in NAc shell. These divergent effects to the GLP‐1R agonists could possibly also be linked to divergent ability of the GLP‐1R agonists to recruit different G‐proteins coupled to intracellular pathways as G‐protein preference studies reveal that Ex4 has biased signalling on the GLP‐1R compared with other agonists.^47^, ^51^, ^52^ One G‐protein preference study comparing Ex4 and liraglutide shows that Ex4 has a higher degree of recruitment of the Gα_i/O_ pathway compared with liraglutide.^47^ Thus, it might explain some degree of variation in neurotransmission;^49^, ^50^ however, the implication of this for the present data is unknown. Future studies are warranted to study the signalling bias of dulaglutide on the GLP‐1R in comparison with Ex4 and liraglutide and its implication on neurotransmission. The divergent outcome associated with the GLP‐1R agonists might also be influenced by factors such as diverse pharmacokinetic profile.^3^, ^4^, ^5^, ^6^ However, as difference in half‐life,^4^, ^5^, ^6^, ^7^, ^9^ administration route^31^ and doses^30^, ^32^(pilot data) were accounted for when selecting the dose‐regimes of Ex4, liraglutide and dulaglutide; these are factors less likely to contribute to the variability in outcomes. On the contrary, variation in distribution volume between Ex4, liraglutide and dulaglutide^4^, ^5^, ^6^, ^7^, ^9^ may potentially contribute to the obtained differences. In addition, factors such as different excipients in the pharmaceutical formulations or deviating affinity towards the GLP‐1R could also influence the obtained data. Upcoming studies should in detail investigate possible properties of Ex4, liraglutide and dulaglutide, at different doses and various administration routes, which may contribute to the obtained variation in behaviour and neurotransmission.

Here, we show a slight reduction in body weight gain by liraglutide in both experimental setups and a tendency in reduction by Ex4 in rats with an acquired skilled reach performance. This corroborates previous studies showing that GLP‐1R agonists reduces the body weight in rats and in humans (for review, see Kanoski et al^13^ and Moreno et al^53^). We further confirm our present observations that GLP‐1R agonist act differently on behaviour per se and that these effects are context‐dependent. The fact that our rats were food‐deprived and had limited access to food could have contributed to the small or lack of effect on bodyweight gain following treatment with various GLP‐1R agonists.

Despite that we here postulate that our data reflect learning and motivation of complex motor skill behaviours, we have to consider that various other factors may influence behaviours investigated in the Montoya staircase test. These include but are not limited to anxiety and stress, which are processes that GLP‐1R agonists alter.^54^, ^55^ The possibility that alterations of gross motor performance influences the outcome in the present study appears less likely because neither Ex4, liraglutide or dulaglutide influences the time spent on the rotarod. Nor does previous studies report that activation of GLP‐1R alters motor behaviour.^22^, ^27^, ^28^, ^29^, ^30^, ^32^ The possibility that GLP‐1R agonists alter the consumption of sucrose pellets by reducing the caloric intake should also be considered. The commonly reported side effect, nausea,^3^, ^6^, ^8^ appears less likely to influence the obtained data as the Ex4‐NAc shell dose and the Ex4‐IP dose do not alter kaolin intake,^20^, ^22^ and the treatment protocols of the various GLP‐1R agonists have been adjusted to reduce the risk of malaise. Supportively, the selected dose and administration route of Ex4, liraglutide or dulaglutide do not alter water intake in rats (pilot data^30^, ^32^). However, conditioned taste aversion may be a confounding factor as systemic injection of Ex4 or liraglutide induces this behaviour.^56^ As it might be difficult to fully dissociate motivation from apparent learning, increased avoidance learning by Ex4 or liraglutide^56^ in rats with an acquired skilled reach performance should be considered as a potential factor contributing to the reduction in the number of pellets consumed. On that note, the increase in performance after dulaglutide treatment to rats during the acquisition of the task might be driven by an increase in motivational processes rather than learning processes. However, this appears very unlikely because GLP‐1R agonists also increases learning processes in other paradigms^16^, ^17^ and decreases operant self‐administration of sucrose and palatable food in rodents.^20^, ^21^ An effect on the number of nonlearning rats could be considered as another potential confounder. However, 39% of the vehicle rats and 33% of the GLP‐1R agonists treated rats were excluded as nonlearners, reducing the possibility that drug treatment affects the number of nonlearners. This number of excluded nonlearners is similar to another study.^26^ Diffusion of Ex4 to surroundings areas might be considered as confounding factors. However, a low volume was used, and previous studies have established that misplaced infusions does not influence behaviours similarly to correct infusions.^57^, ^58^ Sex influence the behaviour outcome of GLP‐1R activation,^59^ and it should be highlighted that the current study only used male rats.

In conclusion, the present data contribute to a further identification of complex behaviours that GLP‐1R agonists modulate. We established that repeated administration of Ex4 or liraglutide reduces the motivation to consume sucrose pellets in rats with an acquired skilled reach performance involving NAc shell dependent neurotransmission. Therefore, present results indicate that these compounds may be beneficial to reduce craving for reinforcing substances in addicted subjects (for review, see Jerlhag^19^). Besides this, we found that dulaglutide enhances the learning of skilled reach behaviours in rats with no prior Montoya staircase experience. The divergent outcomes following Ex4, liraglutide and dulaglutide treatment should be considered when designing upcoming experiments.

## CONFLICT OF INTEREST

The authors have no conflict of interest to declare.

## ETHICAL APPROVAL

The study was approved by the Gothenburg Animal Research Ethics Committee (ethical number: 151‐2015). All experiments were designed and conducted in accordance with the 3R principles.

## FUNDING INFORMATION

The study is supported by grants from the Swedish Research Council (2015‐03219; 2014‐3888; 2019‐01676), Swedish Society for Medical Research, Arvid Carlsson foundation, The Swedish brain foundation, LUA/ALF (Grant 723941) from the Sahlgrenska University Hospital. The funding source had no involvement in data collection, analysis and interpretation of data; in the writing of the report; and in the decision to submit the article for publication.

## AUTHOR CONTRIBUTION

EJ, LA and FB designed the experiment; JV, VL and LA performed the experiments; JV, VL, LA, EJ and FB analysed the data; JV, FB, LA and EJ wrote the paper. All authors contributed to and have approved the final manuscript.

## Supporting information


**Figure S1.** Representative rat brain slice for electrophysiology recordings in A) the nucleus accumbens, (NAc) shell, the dorsomedial striatum (DMS) and the dorsolateral striatum (DLS), and B) the medial prefrontal cortex (mPFC). In brain slices from rat with acquired skilled reach performance, perfusion of exendin‐4 (Ex4), in comparison with vehicle (aCSF) decreases population spike amplitude in C) DMS, D) DLS, E) and mPFC. Data are presented as mean ± SEM; ***P < 0.001. Light grey square represents when drug or aCSFwas perfused onto the slices.
Figure S2. Whole cell recordings show that exendin‐4 (Ex4) perfusion did not alter the A) sIPSCamplitude B) or sIPSCfrequency of nucleus accumbensshell slices in treatment naïve rats and Ex4 did neither alter the C) sIPSCamplitude D) or sIPSCfrequency in rats with an acquired skilled reach performance. Data are presented as mean ± SEM; n.s. = not significant
Figure S3. In rats with a similar acquired skilled reach performance, neither A) repeated exendin‐4 (Ex4), B) repeated liraglutide (Lir), nor C) dulaglutide (Dul) treatment, altered the time at the rotarodcompared to vehicle (Veh). In these three experiments, there were no differences in time at the rotarodat baseline (A‐C). D) There was no difference in baseline time at the rotarod in rats later infused with Ex4 or vehicle into nucleus accumbensshell. Data are presented as mean ± SEM; n.s. = not significant
Figure S4. In rats with an acquired skilled reach performance, the body weight gain was A) not affected by repeated exendin‐4 (Ex4) treatment, B) reduced by repeated liraglutide (Lir) injections, C) was not altered by dulaglutide (Dul) administration and D) was not influenced by local infusion of Ex4 into nucleus accumbensshell. Data are presented as mean ± SEM; ***P < 0.001 and n.s. = not significant
Figure S5. In rats with no prior exposure to the Montoya staircase, neither A) repeated exendin‐4 (Ex4), B) repeated liraglutide (Lir), nor C) dulaglutide (Dul) altered the time at the rotarod (sec) compared to vehicle (Veh). Data are presented as mean ± SEM; n.s. = not significant
Figure S6. Effects of treatment on body weight gain following A) repeated exendin‐4 (Ex4) treatment, B) repeated liraglutide (Lir) injections, or C) dulaglutide (Dul) administration compared to vehicle (Veh). Data are presented as mean ± SEM; n.s. = not significantfor treatment effect in two‐way ANOVA. There is however an overall effect of treatment x time interaction (F(9,126) = 1.99, *P* = 0.045)for Liron body weight gain.

**Table S1.** Schematic description of seven experiments (Exp) that were undertaken in male rats treated with vehicle (Veh), exendin‐4 (Ex4), liraglutide (Lir) or dulaglutide (Dul). In these, rotarod (R) tests and Montoya staircase tests (M) were conducted. *Some rats were euthanised after five days of training in the Montoya test, and were used for subsequent electrophysiological recordings.
Table S2. Coordinates for the NAc shell in rats.
Table S3. Description of the number of excluded non‐learning rats in each experiment conducted. In experiment 1–4 only rats with acquired skilled reach performance were used, allowing exclusion of non‐learning rats prior to drug randomisation. In experiment 5–7 rats without prior experience to the Montoya staircase test were treated throughout the entire test, allowing exclusion of non‐learning rats at the end of the experiment.
Table S4. Baseline group characteristics after stratification and division of rats into treatment groups.

## Data Availability

The data that support the findings of this study are available from the corresponding author upon reasonable request.
