# Supplementary material for: Activation of glucagon‐like peptide‐1 receptors and skilled reach foraging
Source: Addict Biol. 2020 Aug 8;26(3):e12953. doi: 10.1111/adb.12953 (PMC8244104; doi:10.1111/adb.12953)
Supplement: Supplementary file 1 — Figure S1. Representative rat brain slice for electrophysiology recordings in A) the nucleus accumbens, (NAc) shell, the dorsomedial striatum (DMS) and the dorsolateral striatum (DLS), and B) the medial prefrontal cortex (mPFC). In brain slices from rat with acquired skilled reach performance, perfusion of exendin‐4 (Ex4), in comparison with vehicle (aCSF) decreases population spike amplitude in C) DMS, D) DLS, E) and mPFC. Data are presented as mean ± SEM; ***P < 0.001. Light grey square represents when drug or aCSFwas perfused onto the slices. Figure S2. Whole cell recordings show that exendin‐4 (Ex4) perfusion did not alter the A) sIPSCamplitude B) or sIPSCfrequency of nucleus accumbensshell slices in treatment naïve rats and Ex4 did neither alter the C) sIPSCamplitude D) or sIPSCfrequency in rats with an acquired skilled reach performance. Data are presented as mean ± SEM; n.s. = not significant Figure S3. In rats with a similar acquired skilled reach performance, neither A) repeated exendin‐4 (Ex4), B) repeated liraglutide (Lir), nor C) dulaglutide (Dul) treatment, altered the time at the rotarodcompared to vehicle (Veh). In these three experiments, there were no differences in time at the rotarodat baseline (A‐C). D) There was no difference in baseline time at the rotarod in rats later infused with Ex4 or vehicle into nucleus accumbensshell. Data are presented as mean ± SEM; n.s. = not significant Figure S4. In rats with an acquired skilled reach performance, the body weight gain was A) not affected by repeated exendin‐4 (Ex4) treatment, B) reduced by repeated liraglutide (Lir) injections, C) was not altered by dulaglutide (Dul) administration and D) was not influenced by local infusion of Ex4 into nucleus accumbensshell. Data are presented as mean ± SEM; ***P < 0.001 and n.s. = not significant Figure S5. In rats with no prior exposure to the Montoya staircase, neither A) repeated exendin‐4 (Ex4), B) repeated liraglutide (Lir), nor C) dulaglutide (Dul) altered [file ADB-26-e12953-s002.pdf]

Figure S1

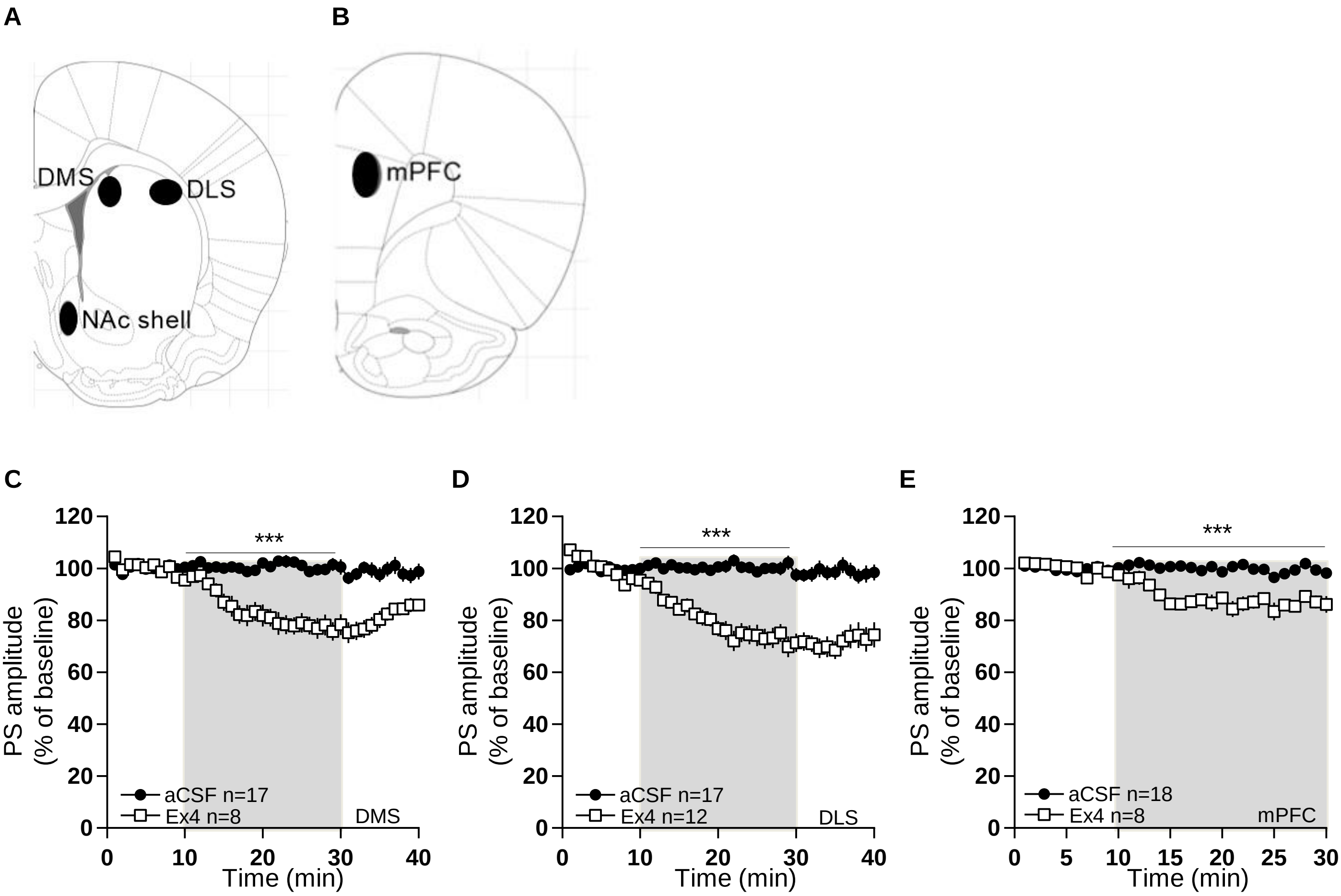

Figure S1. Representative brain slices for electrophysiological recordings and data obtained from perfusion of exendin-4 (Ex4) onto slices from dorsomedial striatum (DMS), dorsolateral striatum (DLS) and medial prefrontal cortex (mPFC).

Representative rat brain slice for electrophysiology recordings in A) the nucleus accumbens, (NAc) shell, the DMS and the DLS, and B) the mPFC. In brain slices from rats with an acquired skilled reach performance, perfusion of Ex4, in comparison with vehicle (aCSF) decreased population spike amplitude in C) DMS, D) DLS, E) and mPFC. Data are presented as mean ± SEM; ***P<0.001. Light grey square represents when drug or aCSF was perfused onto the slices.

Figure S2

Untrained rats

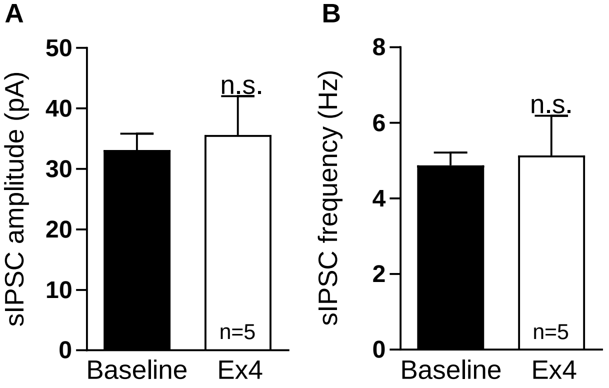

Trained rats

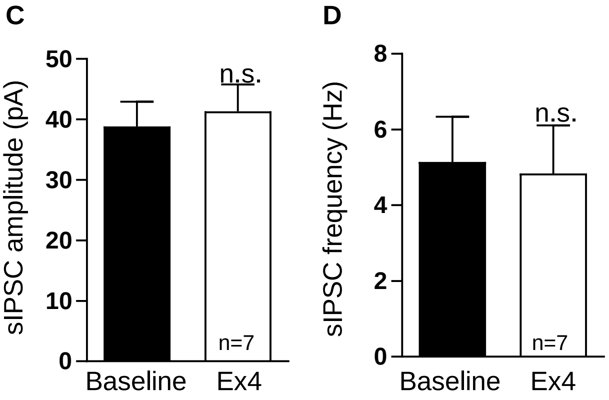

Figure S2. Exendin-4 (Ex4) did not alter the spontaneous inhibitory post-synaptic currents (sIPSC) in nucleus accumbens (NAc) shell slices.
Whole cell recordings show that Ex4 perfusion did not alter the A) sIPSC amplitude B) or sIPSC frequency of NAc shell slices in treatment naïve rats and Ex4 did neither alter the C) sIPSC amplitude D) or sIPSC frequency in rats with an acquired skilled reach performance. Data are presented as mean ± SEM; n.s.= not significant

Figure S3

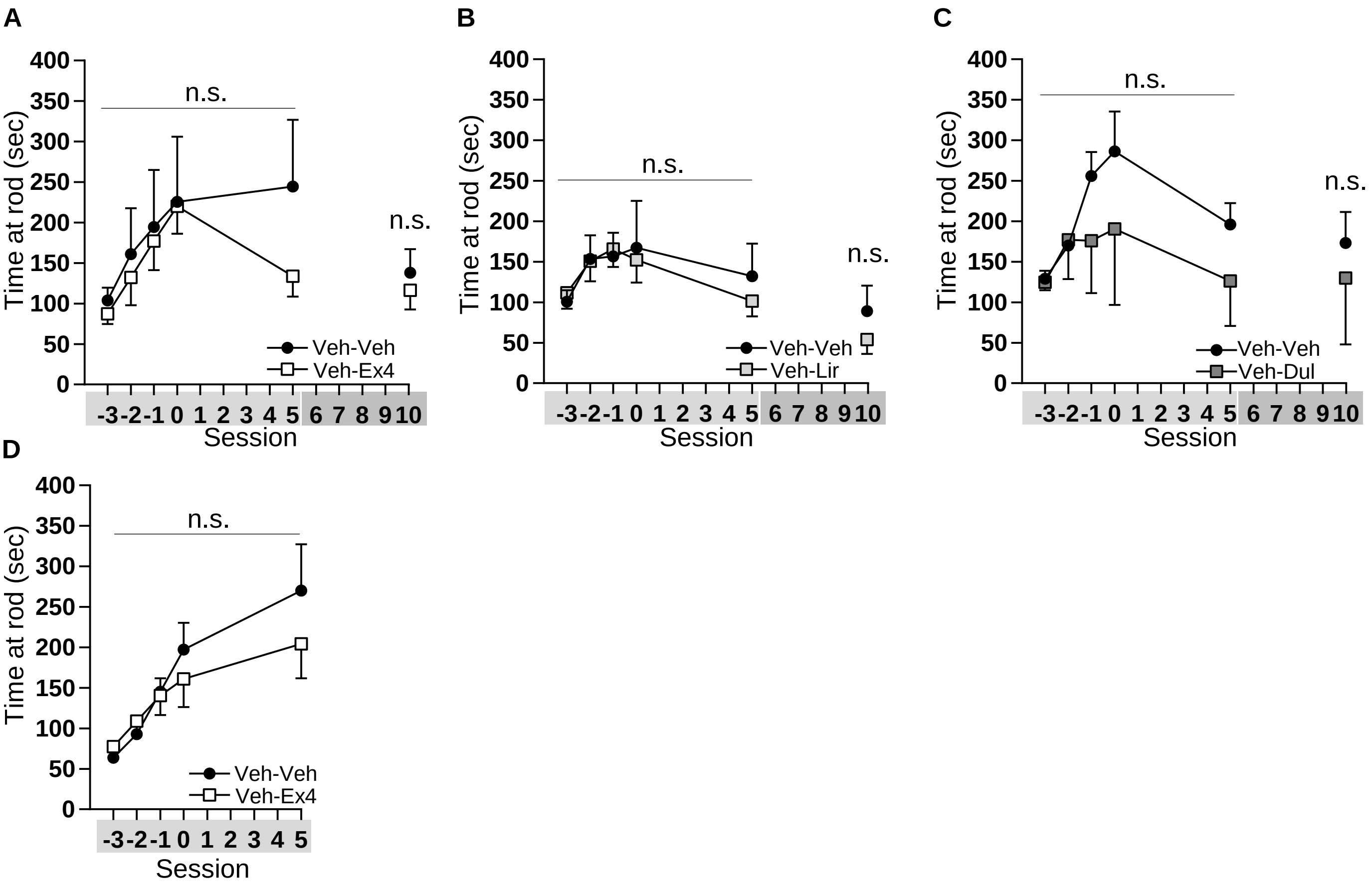

Figure S3. Exendin-4 (Ex4), liraglutide (Lir) or dulaglutide (Dul) did not alter the time at the rotarod in rats with an acquired skilled reach performance.
In rats with a similar acquired skilled reach performance, neither A) repeated Ex4, B) repeated Lir, nor C) Dul treatment, altered the time at the rotarod compared to vehicle (Veh). In these three experiments, there were no differences in time at the rotarod at baseline (A-C). D) There was no difference in baseline time at the rotarod in rats later infused with Ex4 or vehicle into nucleus accumbens shell. Data are presented as mean ± SEM; n.s.= not significant

Figure S4

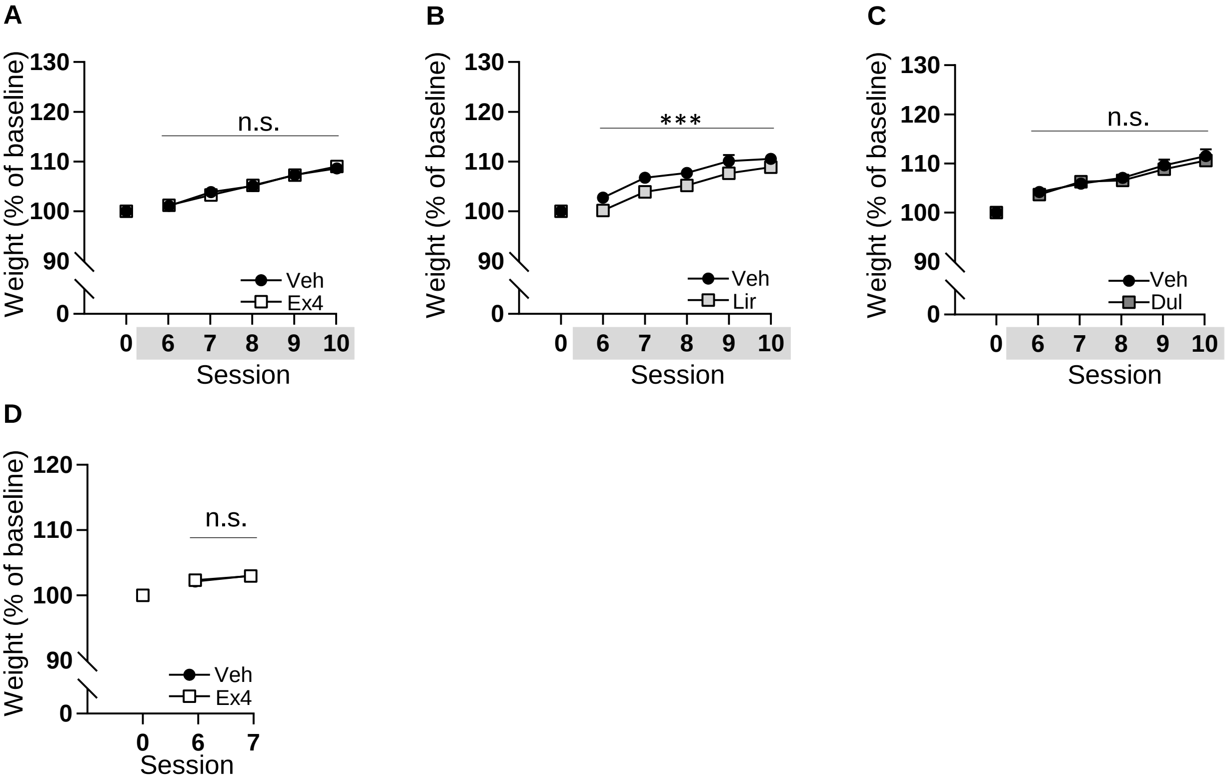

Figure S4. Liraglutide (Lir), but not exendin-4 (Ex4) or dulaglutide (Dul), decreased the body weight gain in rats with an acquired skilled reach performance.
In rats with an acquired skilled reach performance, the body weight gain was A) not affected by repeated Ex4 treatment, B) reduced by repeated Lir injections, C) was not altered by Dul administration and D) was not influenced by local infusion of Ex4 into nucleus accumbens shell. Data are presented as mean ± SEM; ***P<0.001 and n.s.= not significant

Figure S5

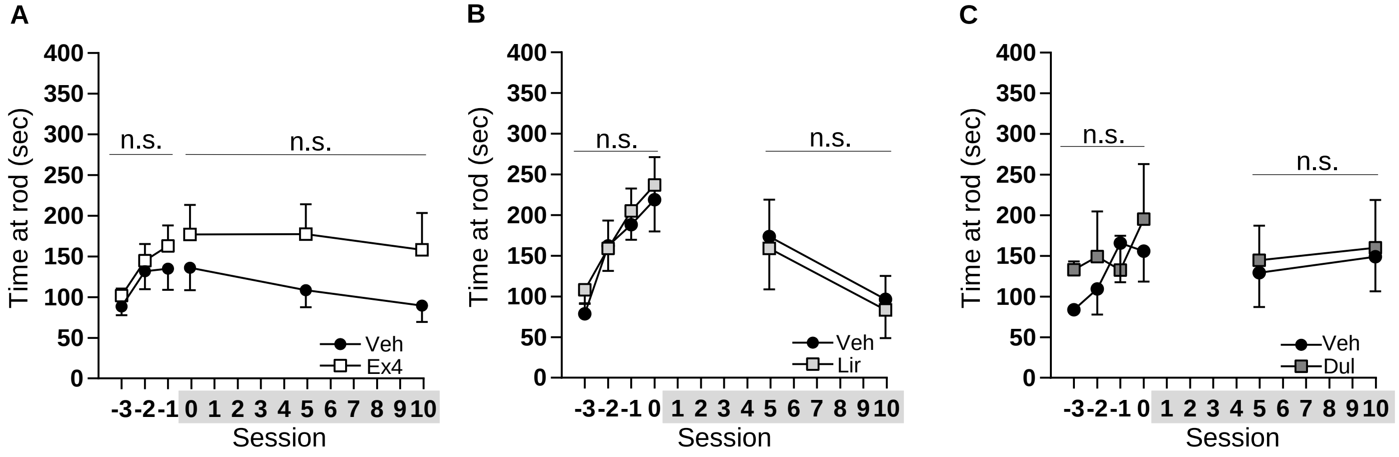

Figure S5. Exendin-4 (Ex4), liraglutide (Lir) or dulaglutide (Dul) did not alter the time at the rotarod in rats with no prior exposure to the Montoya staircase.
In rats with no prior exposure to the Montoya staircase, neither A) repeated Ex4, B) repeated Lir, nor C) Dul altered the time at the rotarod (sec) compared to vehicle (Veh). Data are presented as mean ± SEM; n.s.= not significant

Figure S6

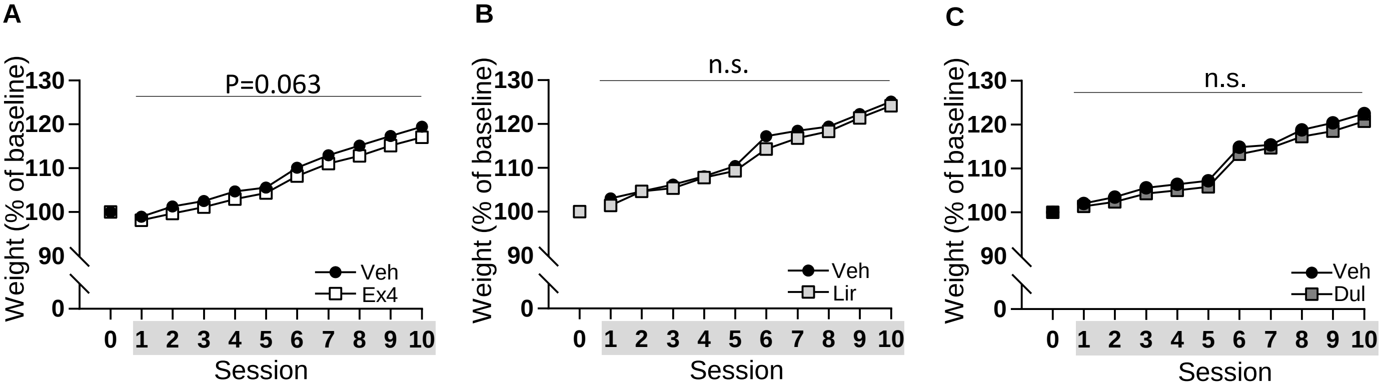

Figure S6. Effects of exendin-4 (Ex4), liraglutide (Lir) and dulaglutide (Dul) on body weight gain.
Effects of treatment on body weight gain following A) repeated Ex4 treatment, B) repeated Lir injections, or C) Dul administration compared to vehicle (Veh). Data are presented as mean ± SEM; n.s.= not significant for treatment effect in two-way ANOVA. There is however an overall effect of treatment x time interaction ($F(9,126)=1.99$, $P=0.045$) for Lir on body weight gain.
