# Supplementary material for: Activation of glucagon‐like peptide‐1 receptors and skilled reach foraging
Source: Addict Biol. 2020 Aug 8;26(3):e12953. doi: 10.1111/adb.12953 (PMC8244104; doi:10.1111/adb.12953)
Supplement: Supplementary file 2 — Table S1. Schematic description of seven experiments (Exp) that were undertaken in male rats treated with vehicle (Veh), exendin‐4 (Ex4), liraglutide (Lir) or dulaglutide (Dul). In these, rotarod (R) tests and Montoya staircase tests (M) were conducted. *Some rats were euthanised after five days of training in the Montoya test, and were used for subsequent electrophysiological recordings. Table S2. Coordinates for the NAc shell in rats. Table S3. Description of the number of excluded non‐learning rats in each experiment conducted. In experiment 1–4 only rats with acquired skilled reach performance were used, allowing exclusion of non‐learning rats prior to drug randomisation. In experiment 5–7 rats without prior experience to the Montoya staircase test were treated throughout the entire test, allowing exclusion of non‐learning rats at the end of the experiment. Table S4. Baseline group characteristics after stratification and division of rats into treatment groups. [file ADB-26-e12953-s001.docx]

**Table S1. Schematic description of seven experiments (Exp) that were undertaken in male rats treated with vehicle (Veh), exendin-4 (Ex4), liraglutide (Lir) or dulaglutide (Dul).**

In these, rotarod (R) tests and Montoya staircase tests (M) were conducted. *Some rats were euthanised after five days of training in the Montoya test, and were used for subsequent electrophysiological recordings.

**Session**

**/Exp**

|  | **-3** | **-2** | **-1** | **0** | **1** | **2** | **3** | **4** | **5** | **6** | **7** | **8** | | **9** | **10** | **11** | **12** |
| --- | --- | --- | --- | --- | --- | --- | --- | --- | --- | --- | --- | --- | --- | --- | --- | --- | --- |
| **1 /**  **Fig. 1** |  | | | Veh | | | | | | Veh/Ex4 | | | | | |  | |
|  |  |  |  |  | M | M | M | M | M* | M | M | M | | M | M |  | |
|  | R | R | R | R |  |  |  |  | R |  |  |  | |  | R |  | |
| **2 /**  **Fig. 1** |  | | | Veh | | | | | | Veh /Lir | | | | | |  | |
|  |  |  |  |  | M | M | M | M | M* | M | M | M | | M | M |  |  |
|  | R | R | R | R |  |  |  |  | R |  |  |  | |  | R |  |  |
| **3 /**  **Fig. 1** |  | | | Veh | | | | | | Veh/Dul | | | | | | Veh/Ex4 | |
|  |  |  |  |  | M | M | M | M | M* | M | M | M | | M | M | M | M |
|  | R | R | R | R |  |  |  |  | R |  |  |  | |  | R |  |  |
| **4 /**  **Fig. 2** |  | | | Veh | | | | | | Veh/Ex4 | | |  | | | | |
|  |  |  |  |  | M | M | M | M | M | M | M |  | | | |  | |
|  | R | R | R | R |  |  |  |  | R |  |  |  |  |  |  |  | |
| **5 /**  **Fig. 3** |  | | | Veh/Ex4 | | | | | | | | | | | |  | |
|  |  |  |  |  | M | M | M | M | M | M | M | M | | M | M |  | |
|  | R | R | R | R |  |  |  |  | R |  |  |  | |  | R |  | |
| **6 /**  **Fig. 3** |  | | |  | Veh /Lir | | | | | | | | | | |  | |
|  |  |  |  |  | M | M | M | M | M | M | M | M | | M | M |  |  |
|  | R | R | R | R |  |  |  |  | R |  |  |  | |  | R |  | |
| **7 /**  **Fig. 3** |  | | |  | Veh/Dul | | | | | | | | | | |  | |
|  |  |  |  |  | M | M | M | M | M | M | M | M | | M | M |  | |
|  | R | R | R | R |  |  |  |  | R |  |  |  | |  | R |  |  |

**Table S2. Coordinates for the nucleus accumbens (NAc) shell in rats.**

| NAc shell coordinates | Distance in mm |
| --- | --- |
| AP | +1.85 |
| ML | ±1.0 |
| DV | -7.8 |
| Extension from guide | 6.8 |

anterior-posterior (AP), medial-lateral (ML), dorsal-ventral (DV).

**Table S3. Description of the number of excluded non-learning rats in each experiment conducted.**

In experiment 1-4 only rats with an acquired skilled reach performance were used, allowing exclusion of non-learning rats prior to drug randomisation. In experiment 5-7 rats without prior experience to the Montoya staircase test were treated throughout the entire test, allowing exclusion of non-learning rats at the end of the experiment.

| Experiment | Number of excluded non-learning rats before drug randomisation | Number of excluded non-learning rats in the GLP-1R agonist treatment group | Number of excluded non-learning rats in the vehicle group |
| --- | --- | --- | --- |
| 1 | 9 |  |  |
| 2 | 16 |  |  |
| 3 | 14 |  |  |
| 4 | 27 |  |  |
| 5 |  | 4 | 9 |
| 6 |  | 5 | 3 |
| 7 |  | 5 | 4 |

**Table S4. Baseline group characteristics after stratification and division of rats into treatment groups.**

| Experiment | Number of pellets consumed (Montoya staircase) | Success rate (Montoya staircase) | Time at the rod  (Rotarod) |
| --- | --- | --- | --- |
| 1 | treatment F(1,25)=0.0004, P=0.984, time F(4,25)=6.51, P=0.001, interaction F(4,25)=0.09, P=0.984 | treatment F(1,25)=0.03, P=0.865, time F(4,25)=3.69, P=0.017, interaction F(4,25)=0.02, P=0.999 | treatment F(1,25)=0.91, P=0.350, time F(4,25)=2.77, P=0.050, interaction F(4,25)=0.26, P=0.900 |
| 2 | treatment F(1,30)=0.09, P=0.762, time F(4,30)=3.33, P=0.023, interaction F(4,30)=0.03, P=0.998 | treatment F(1,30)=0.46, P=0.503, time F(4,30)=3.90, P=0.012, interaction F(4,30)=0.07, P=0.991 | treatment F(1,30)=0.08, P=0.777, time F(4,30)=1.35, P=0.276, interaction F(4,30)=0.16, P=0.955 |
| 3 | treatment F(1,20)=0.12, P=0.736, time F(4,20)=5.11, P=0.005, interaction F(4,20)=0.10, P=0.981 | treatment F(1,20)=0.0005, P=0.942, time F(4,20)=4.14, P=0.013, interaction F(4,20)=0.65, P=0.633 | treatment F(1,20)=2.42, P=0.136, time F(4,20)=1.86, P=0.157, interaction F(4,20)=0.45, P=0.772 |
| 4 | treatment F(1,45)=0.13, P=0.720, time F(4,45)=6.38, P<0.001, interaction F(4,45)=0.58, P=0.680 | treatment F(1,45)=0.64, P=0.428, time F(4,45)=2.51, P=0.055, interaction F(4,45)=0.88, P=0.481 | treatment F(1,45)=0.51, P=0.481, time F(4,45)=13.68, P<0.001, interaction F(4,45)=0.53, P=0.711 |
| 5 | Not applicable | Not applicable | treatment F(1,23)=0.49, P=0.490, time F(2,46)=11.53, P<0.001, interaction F(2,46)=0.26, P=0.772 |
| 6 | Not applicable | Not applicable | treatment F(1,14)=0.11, P=0.748, time F(3,42)=13.22, P<0.001, interaction F(3,42)=0.18, P=0.908 |
| 7 | Not applicable | Not applicable | treatment F(1,13)=0.27, P=0.613, time F(3,39)=1.83, P=0.159, interaction F(3,39)=0.817, P=0.493 |
